# Regulation of the Postsynaptic Compartment of Excitatory Synapses by the Actin Cytoskeleton in Health and Its Disruption in Disease

**DOI:** 10.1155/2016/2371970

**Published:** 2016-04-05

**Authors:** Holly Stefen, Chanchanok Chaichim, John Power, Thomas Fath

**Affiliations:** ^1^Neurodegeneration and Repair Unit, School of Medical Sciences, University of New South Wales, Sydney, NSW 2052, Australia; ^2^Translational Neuroscience Facility, School of Medical Sciences, University of New South Wales, Sydney, NSW 2052, Australia

## Abstract

Disruption of synaptic function at excitatory synapses is one of the earliest pathological changes seen in wide range of neurological diseases. The proper control of the segregation of neurotransmitter receptors at these synapses is directly correlated with the intact regulation of the postsynaptic cytoskeleton. In this review, we are discussing key factors that regulate the structure and dynamics of the actin cytoskeleton, the major cytoskeletal building block that supports the postsynaptic compartment. Special attention is given to the complex interplay of actin-associated proteins that are found in the synaptic specialization. We then discuss our current understanding of how disruption of these cytoskeletal elements may contribute to the pathological events observed in the nervous system under disease conditions with a particular focus on Alzheimer's disease pathology.

## 1. Introduction

Memories are coded in the ensemble activity of small groups of neurons distributed throughout the brain. Glutamate is the primary excitatory neurotransmitter in the brain and the majority of synaptic connections between the glutamatergic neurons are made on dendritic spines. These specialized dendritic protrusions are supported by an actin-rich cytoskeletal protein matrix that not only provides structural support but also is essential for the delivery and anchoring of neurotransmitter receptors and other molecules involved in synaptic transmission. The synapse's capacity for change allows for memory formation and adaption to the environment. This synaptic remodelling is a dynamic process involving trafficking of neurotransmitter receptors into or out of the synaptic complex. These modifications require regulated disassembly and reassembly of the actin cytoskeleton. Orchestrating the controlled breakdown and reassembly of the actin cytoskeleton requires coordinated activity of an array of actin-associated proteins.

Alzheimer's disease (AD) is a neurodegenerative brain disorder that erodes memories and clouds thinking, gradually destroying one's sense of self. A loss of synaptic connectivity is thought to underlie the cognitive symptoms of AD. Synapse loss is observed in early stages of the pathology [1] and the correlation between synapse loss and severity of cognitive impairment is well established [2–4]. The early emergence of altered network connectivity has been confirmed by subsequent functional imaging studies [5, 6].

Cellular and murine models of AD have provided insight into the cellular mechanisms that underlie the loss of synaptic function in AD. It has become increasingly apparent that actin cytoskeletal function is disrupted in the pathology. Here we review the literature, describing the contribution of actin-associated proteins to synaptic function, and highlight recent findings implicating their involvement in AD pathology. Given the central role of the actin cytoskeleton in maintaining and modifying glutamatergic synaptic connections, proteins that modify or stabilize the cytoskeletal structures are potential therapeutic targets in the treatment of AD.

## 2. Structural and Functional Organization of the Postsynaptic Compartment of Excitatory Synapses

The majority of synaptic contacts between excitatory neurons are made on dendritic spines. These small structures house the postsynaptic molecules necessary for synaptic transmission. The prototypical spine contains a bulbous head (0.01–1 *μ*m^3^) and is connected to its parent dendrite via a thin (0.1 *μ*m diameter) spine neck [7], which restricts diffusion between the two compartments, allowing concentration and segregation of signalling molecules [8, 9]. At the distal end of the spine head, directly across the synaptic cleft from the active zone of the presynaptic bouton, is an electron-dense postsynaptic density (the PSD), within which neurotransmitter receptors, cell adhesion molecules, cell signalling molecules, and a myriad of molecules involved in synapse stability are embedded [10, 11].

Spines are diverse in both shape and intracellular constituents. The neck length and width and head size vary along a continuum even within the same section of dendrite [12]. Despite this continuum they are usually classified based on the relative size of the spine head and neck [13, 14]. Mushroom spines have a large head and thin neck. Thin spines have a long neck and small head. Stubby spines are short with no obvious neck constriction. Synaptic function and structure are tightly intertwined and the different shapes are thought to reflect differences in synaptic strength and developmental stage [15–18]. Spine head volume has been found to be tightly correlated with PSD area [19] and the number of *α*-amino-3-hydroxy-5-methyl-4-isoxazolepropionic acid receptors (AMPARs) in the postsynaptic membrane [20]. Thus, spines with largest heads form the strongest synaptic connections. These mushroom spines are less motile and more persistent than thin spines [21–23]. Large spines are also more likely to contain organelles including endoplasmic reticulum and mitochondria [24], likely due to increased metabolic demands associated with maintaining the expanded synaptic machinery.

The morphology of dendritic spines is highly dynamic. Spines undergo functional and morphological changes during development and in response to neuronal activity. Nascent spines emerge as thin or filopodia-like protrusions [23, 25, 26]. Most of these newly formed spines will disappear, whereas others that find synaptic partners will undergo a morphological transition into the more stable “mushroom” spines [26]. Once established, spines continue to be sculpted by neuronal activity. As discussed above, spine morphology and function are linked with the spine head volume tightly correlated with the number of AMPARs in the postsynaptic membrane. Therefore, the trafficking of neurotransmitter receptors to the plasma membrane is essential for synapse maturation and activity-dependent changes in synapse strength thought to underlie memory formation. Note that memories and their synaptic substrates can persist for years, well beyond the lifetime of the proteins responsible for synaptic transmission. Thus, even synapse maintenance requires cycling of proteins in and out of the membrane for protein replenishment.

The synaptic cytoskeleton not only is necessary for the structural support of the synaptic connections but also is critical for the cycling of neurotransmitter receptor and other proteins between the plasma membrane and endoplasmic compartment. The primary cytoskeletal component found in dendritic spines is actin [27]. Actin is present in two forms: filamentous actin (F-actin), which is the insoluble, polymer form that makes up the cytoskeleton, and its soluble monomeric building block, globular actin (G-actin). F-actin in spines displays a compartment specific organization with more linear oriented actin filaments in the spine neck and a branched organization in the head compartment [28].

Time-lapse studies suggest that spines initially develop as filopodia, which grow the mushroom-shaped head, characteristic of mature spines through branching of actin filaments (comprehensively reviewed by Yoshihara et al. [26] and Hotulainen and Hoogenraad [29]). These studies also highlight the dynamic nature of the actin cytoskeleton. Spine shape changes follow changes in actin dynamics and have been observed occurring within seconds [30], responding to chemical or electrical stimulation [31]. Live-cell imaging of green fluorescent protein labelled actin (GFP-actin) has indicated that actin is organized into structurally and functionally distinct F-actin populations within the postsynaptic compartment [15, 30]. Dynamic and stable pools of F-actin were identified that consisted of differing rates of actin treadmilling [30]. The dynamic pool of F-actin was shown to have an actin turnover rate <1 min and is believed to be involved in generating force to expand the spine head and AMPAR insertion at the PSD. A stable pool of F-actin was localized to the base of the spine and had a slower turnover rate of approximately 17 min. The stable pool was suggested to provide resistance against the force generated by the active pool, maintaining the stability of the spine [15]. Honkura and colleagues also identified a third pool of F-actin, an enlargement pool, which was required for spine head enlargement during the induction of long-term potentiation (LTP).

A major challenge in the analysis of structural and functional changes at synapses is the extremely small size, with the spine heads being less than a micron in diameter. Advances in superresolution microscopy provide us with some fundamental understanding of actin dynamics in dendritic spines. Photoactivated localization microscopy (PALM) revealed a greater velocity of actin movement in the spine head and a net flow of G-actin from dendrite into the spine [32]. Stimulated emission depletion (STED) imaging shows not only size changes, but also small shape changes that are difficult to detect with other methods. Stimulation often causes spines to take on a cup-like shape [33].

In more recent years, superresolution imaging has been implemented in various studies to more accurately examine dendritic spine properties such as morphology and diffuse dynamics of proteins, calcium, and small molecules [34]. For instance, Lu and colleagues [35] were able to visualise single molecule dynamics of the actin cytoskeleton modulating kinase calcium/calmodulin dependent kinase II (CAMKII) [36] within dendritic spines by using PALM. By implementing this high resolution technique Lu and team were able to distinguish multiple subpopulations of CAMKII within the spine head based on motility. Furthermore, STED imaging in combination with fluorescence recovery after photobleaching (FRAP) was able to show that, upon stimulation of spine-specific LTP, as spine heads become larger, spine necks become shorter and wider [37]. In addition to this finding, Tønnesen and colleagues showed that spines which appear stubby in two-photon imaging are mushroom headed with short necks when visualised with STED. These studies highlight the importance of using superresolution imaging techniques when investigating characteristics of spines.

## 3. Regulation of the Postsynaptic Actin Cytoskeleton

The actin cytoskeleton in eukaryotic cells is regulated by a host of actin-associated proteins. The complex actions of these proteins govern actin cytoskeleton dynamics, enabling functional and structural diversity of F-actin populations within dendritic spines. Actin regulators facilitate assembly, disassembly, branching, stabilization, and reorganization of the cytoskeleton, all critical requirements for synaptic plasticity.

Assembly of actin filaments and as such the actin cytoskeleton requires the formation of rod-like actin polymers known as actin filaments or filamentous actin (F-actin) from actin monomers (globular actin, G-actin). The process by which G-actin is accommodated into the fast growing (barbed) end of an actin filament and dispersed from the opposite pointed end is referred to as actin treadmilling. Various actin-associated proteins are involved in regulating the assembly of actin filaments. In the following section we will focus our attention on a select number of key regulators, including the actin sequestering protein profilin, the actin nucleators formin and actin-related proteins 2 and 3 complex (Arp2/3), the actin depolymerizing factor (ADF)/cofilin, the actin motor protein myosin, and the actin stabilizing protein tropomyosin.

### 3.1. Actin Sequestering and Nucleating Proteins

Actin filament nucleation can occur* de novo* or as filament branches that nucleate on preexisting filaments. Formins are a superfamily of proteins with at least 15 different proteins found in mammalian cells that promote the* de novo* nucleation of unbranched actin filaments (for reviews, see [38, 39]). Their activity is regulated by small GTPases thereby controlling the assembly of new actin filaments [40–42]. Formins play a critical role in supporting the early morphogenesis of filopodial spines [43], and it localizes to fine, filopodial structures that are found at the distal part of more mature spines [44].

Arp2/3 promotes nucleation of F-actin daughter branches of existing F-actin mother filaments [45]. Actin filaments within filopodia were found to originate from branch points in lamellipodia that were generated by Arp2/3 [46]. Arp2/3 complex is detected in the central region of the spine head approximately 200–400 nm from the PSD indicating a local segregation of morphologically distinct actin filament populations [47].

Depletion of Arp2/3 complex in both B35 neuroblastoma cells and primary hippocampal neurons was found to decrease growth cone F-actin and reduce lamellipodia protrusion and contraction [46]. In addition to this, cells with deficient levels of Arp2/3 had lamellipodia that were narrower and contained actin networks that were less complex and contained fewer branches [46]. Conversely, in a study by Yang and colleagues [48], inhibition of Arp2/3 using the reversible Arp2/3 inhibitor CK-666 unexpectedly resulted in an increase in actin retrograde flow, which was significantly reduced upon inhibition of myosin II, suggesting that Arp2/3 restricts myosin II mediated retrograde flow of actin [48].

Activation of Arp2/3 occurs via the activity of nucleation promoting factors (NPFs) such as neural Wiskott-Aldrich syndrome (N-WASP), WASP family verprolin-homologous protein (WAVE, also known as SCAR), and WASP and SCAR homolog (WASH) [49]. Arp2/3 and WASH have been implicated in early endosome morphology and function. Through immunocytochemical analysis of fibroblast-like cells, WASH was found to extensively associate with early endosome markers EEA1 and Rab5 and weakly associate with recycling endosome marker Rab11 [50]. siRNA-mediated knockdown of WASH resulted in larger and more elongated EEA1 positive structures compared to controls [50]. Knockdown of WASH reduced trafficking of epidermal growth factor (EGF) to late endosomes, an effect also observed in response to actin-polymerization disruption. However, knockdown of WASH was not found to affect reuptake or recycling of transferrin, implying specificity of WASH to the endocytic degradation pathway [50]. These results suggest that WASH activity affects endosomal trafficking of cargo, most likely via Arp2/3 mediated actin dynamics.

Profilin is thought to promote F-actin elongation at the barbed end by accelerating the nucleotide exchange of ADP for ATP on G-actin. Recent findings suggest that profilin has separable roles in G-actin regulation [51]. Suarez and colleagues found that profilin is required for actin contractile ring formation in fission yeast through interactions with formin as well as limiting Arp2/3-mediated actin branching by sequestering G-actin [51]. Profilin is thought to favour formin mediated F-actin elongation over Arp2/3 while reducing the ability of both formin and Arp2/3 to nucleate filaments [51].

### 3.2. Actin Filament Depolymerizing Proteins

Turnover and disassembly of F-actin occur through the actions of ADF/cofilin. With the continual addition of monomeric ATP-bound actin to the barbed end of the filament, previously incorporated actin monomers become progressively distal to the barbed end to form the body of the filament. As this occurs, the ATP is hydrolysed to ADP [52]. The resulting ADP-bound actin subunits are still able to maintain filament stability due to the presence of an inorganic phosphate (P_(i)_). It is believed that once P_(i)_ has been released cofilin is able to bind to the filament, inducing severing and depolymerization [53].

Severing of ADP-actin from the pointed end of filaments is facilitated by the actions of ADF/cofilin in its dephosphorylated active state [54]. ADF/cofilin interacts directly with actin filaments, and its activity is regulated by the actions of LIM-Kinase 1 (LIMK-1) and Slingshot (SSH) phosphatase. Phosphorylation of cofilin at its serine 3 site by LIMK-1 inhibits ADF/cofilin severing of actin filaments and increases F-actin content in actin-rich regions of neurons [55, 56]. Conversely, dephosphorylation as well as activation of cofilin by SSH results in severing of actin filaments. Based on cryoelectron microscopy three-dimensional reconstructions of cofilin bound to actin filaments, Galkin and colleagues [57] postulate that filaments decorated with cofilin undergo a conformational change whereby the actin protomers bound to cofilin rotate in a manner that induces greater flexibility of the filament. Once rotated, the filament exposed regions that were then vulnerable to severing through further ADF/cofilin actions [57]. The ability of ADF/cofilin to disassemble F-actin networks has been suggested to be integral to the enlargement of dendritic spine heads, possibly by creating new barbed ends from severed filaments [58]. The activity of cofilin can be modified by upstream signalling proteins such as Cdc42, which has been shown to promote cofilin activation [59].

Gelsolin is an actin-associated protein predominantly activated by Ca^2+^ [60]. When activated by Ca^2+^, gelsolin undergoes conformational changes that expose actin binding sites [60–62]. Active gelsolin severs and then caps F-actin at barbed ends, resulting in the disassembly of the F-actin network and prevention of further polymerization [63–65]. However, in the presence of phosphatidylinositol 4,5-bisphosphate (PIP_2_), gelsolin activity is largely abolished [66]. Furthermore, a study by Hartwig and colleagues [67] found that inhibition of gelsolin by PIP_2_ application increased the prevalence of barbed ends suggesting that PIP_2_ facilitates the removal of gelsolin caps from F-actin barbed ends. With the inhibition of gelsolin, further polymerization of actin filaments is enabled [68]. Overexpression of gelsolin in a PC12 neuronal-like cell line differentiated with nerve growth factor led to an increase in neurite length and motility rate compared to controls possibly through increased F-actin turnover [69]. Importantly, gelsolin function is required for the morphological transition of filopodia to spines [70].

### 3.3. Actin Stabilizing Proteins and “Gatekeepers” of Actin Filament Dynamics and Stability

Drebrin A (DA) is the adult isoform of drebrin and is found in mature neurons at postsynaptic sites of dendritic spines [71]. Drebrin A binds to F-actin, inhibiting depolymerization of the filament predominantly at the barbed end [72, 73]. An atomic force spectroscopy study by Sharma and colleagues [74] found that drebrin binding resulted in a twisting of F-actin conformation that induced stiffening of the filament. The conformational twist induced by drebrin was found to occur in a manner that was opposite to cofilin induced twisting in F-actin structure, suggesting that drebrin and cofilin have antagonistic effects of F-actin structure [74].

Drebrin has been shown to compete with cofilin for F-actin binding sites [75]. Cosedimentation experiments showed that DA and cofilin are able to simultaneously bind to F-actin and inhibit the actions of each other [75]. Neurons transfected with DA-GFP were found to have dendritic spines that had significantly lengthened necks compared to controls [71]. Binding of drebrin to F-actin was also shown to inhibit myosin V binding.* In vitro* motility assays showed an impairment of F-actin binding to myosin V coated glass surfaces when in the presence of drebrin A [72]. However, F-actin that was able to bind to myosin V in the presence of drebrin did not show any impairment in F-actin-myosin sliding. This suggests that drebrin may also modulate myosin V activity.

Tropomyosins (Tpms) are a family of actin-associated proteins and key regulators of the actin cytoskeleton. In mammals, TPM1, TPM2, TPM3, and TPM4 genes have been shown to be responsible for the generation of more than 40 known tropomyosin isoforms [76] with gene products from TPM1, TPM3, and TPM4 found in neuronal cells. The primary structure of Tpm proteins consists of paired *α*-helices arranged in a coiled-coil manner [77, 78]. Although the primary structure of tropomyosin is highly conserved between the various isoforms, alternatively spliced exons allow functional diversity and differential localization within the cell [76, 79, 80].

Studies that have examined the interaction between Tpm and actin filaments have shown that Tpm isoforms have distinct F-actin regulatory effects as well as differential affinities to associate with F-actin [76, 78, 81] and facilitating functional diversity of cytoskeletal F-actin [82]. Tpm3.1, a tropomyosin isoform derived from the TPM3 gene, is involved in F-actin stabilization and reduced cell motility [76, 82] whereas Tpm1.12, derived from the TPM1 gene, promotes F-actin-ADF interactions resulting in F-actin severing [76].

Further studies exploring the effects of increased Tpm3.1 protein levels found an increase in the length and branching of dendrites and axons, along with increased growth cone size [83] and an increased pool of filamentous actin in growth cones [84]. A subsequent study showed altered growth cone dynamics in response to the knockdown of Tpm3.1 and Tpm3.2 [85]. These results suggest that this particular tropomyosin isoform may be involved in the stabilization of the actin cytoskeleton in neurons. Guven and colleagues [79] have detected Tpm3 gene products in the postsynaptic compartment of mature cultured neurons suggesting a potential role in the maintenance of synaptic connections.

Another mode of actin filament regulation and stabilization is through actin capping proteins, which bind to the barbed end of actin, preventing further elongation. This process limits the length of actin filaments. Actin capping protein (CP) has been found to be associated with the actin and Arp2/3 network in spine heads [28], and the level has been observed to be elevated during stages of synapse formation. Knockout of CP in rat hippocampal cultures has been shown to lead to altered spine morphology and a reduction in synapse density [86]. Another protein with actin capping function, Eps8, has been found to play a role in actin-based motility, such as filopodia growth and numbers [87]. Eps8 is also enriched in the PSD, and as in the case of CP knockdown, reduced expression levels alter spine morphology [88]. The actin severing protein gelsolin can also cap actin filaments, depending on calcium ion concentration [89]; however this has not been studied in detail in neurons.

### 3.4. Actin Motor Proteins

Myosins form a superfamily of actin motor proteins. The family contains motors with diverse functions that range from building contractile elements (conventional family members) such as the sarcomere of muscle cells to driving intracellular transport of vesicles (unconventional family members). Characteristic to all myosins is the presence of catalytic head or motor domains that bind and hydrolyse ATP to produce motility. The tail of myosins can either align with other myosin molecules to form myosin filaments (e.g., the formation of the thick filaments in muscle, built by muscle isoforms of myosin II) or bind to different cargos allowing transport of these cargos along actin filaments. For a review on myosin function and diversity, see Hartman and Spudich [90]. The motor domains of the myosins bind to F-actin [91] and have been shown to travel along the filament in a hand-over-hand type movement using various imaging techniques [92–94]. The unconventional myosins V and VI have been implicated in vesicle trafficking within dendritic spines [95, 96].

Together these actin-associated proteins provide diverse regulation of the cytoskeleton within the postsynaptic compartment of dendritic spines.

## 4. Learning and Memory as Actin Cytoskeleton-Dependent Process

Long-term changes in connections between neurons are thought to be the basis of memory formation. At most synapses in the brain, activity-dependent synaptic plasticity is triggered by a rise in postsynaptic calcium, which triggers a series of downstream effectors that can initiate different forms of synaptic plasticity, including long-term potentiation (LTP) and long-term depression (LTD) of synaptic transmission [97]. Although the loci of expression can vary between pre- and postsynaptic structures depending on the synapse, developmental stage, and induction protocol, at forebrain glutamatergic synapses synaptic plasticity often manifests as a change in the number of AMPA receptors expressed at the synapse [98].

As discussed above, synaptic proteins are continuously shuttled in and out of the plasma membrane; thus the number of AMPARs at the synapse is governed by the relative rate of receptor exocytosis and endocytosis, resulting in concomitant spine head volume changes. Several studies have shown that LTP is accompanied by an increase in spine volume [22, 31, 99] whereas LTD is accompanied by a reduction in spine volume [31, 100].

Actin dynamics are integral to both structural and functional synaptic plasticity. The LTP-associated spine enlargement is associated with an increase in F-actin [101] that persisted for several weeks. Furthermore, blocking actin polymerization via application of latrunculin and cytochalasin toxins impairs LTP and spine enlargement [102–106]. Conversely, LTD is associated with a relative decrease in F-actin [31] and stabilization of the actin cytoskeleton impairs LTD and the associated reduction in spine volume [107]. Application of drugs impacting actin stability also disrupts a variety of associative learning tasks (see [108] for review).

Measurements of fluorescence recovery of photobleached green fluorescent protein-tagged actin (GFP-actin) in the spine head indicate that most of the actin in the spine head is found in filaments that rapidly turn over, with only a small fraction of actin being assembled in the form of stable filaments [30]. Actin dynamics are altered by neuronal activity. High frequency stimulation, which induces LTP and enlarges the spine head, is associated with an increase in proportion of actin present as F-actin in the spine head [31]. Conversely, LTD-inducing low-frequency stimulation was found to reduce the spine head volume and the F-actin : G-actin ratio [31].

Using photoactivatable GFP-actin, Honkura and colleagues [15] tracked the spatial temporal movement of actin. Their data provide evidence for three functional pools of F-actin: a dynamic pool at the tip of the spine head, a stable pool at the base of the spine, and an “enlargement pool” that emerges following repeated stimulation of the spine head with glutamate. The persistence of the enlargement pool in the spine head was associated with structural LTP.

In addition to actin, several actin-associated molecules show activity-dependent changes in phosphorylation state and distribution. LTP induces a transient increase in spines immunopositive for phosphorylated (deactivated) cofilin [109]. A recent study by Bosch et al. [110] describes the spatiotemporal dynamics of GFP tagged actin and several GFP tagged actin-associated proteins following the induction of LTP. They found that structural LTP was associated with the rapid translocation of actin and several actin-associated proteins into the spine head. Of particular note was cofilin, which became concentrated in the spine head.

Structural plasticity is not limited to the neurons. Actin-rich perisomatic astrocytic processes [111] at glutamatergic synapses show activity-dependent remodelling, expanding in concert with spine enlargement following the induction of LTP [112].

## 5. The Actin Cytoskeleton as Key Regulator in Glutamate Receptor Trafficking

**Figure 1:**
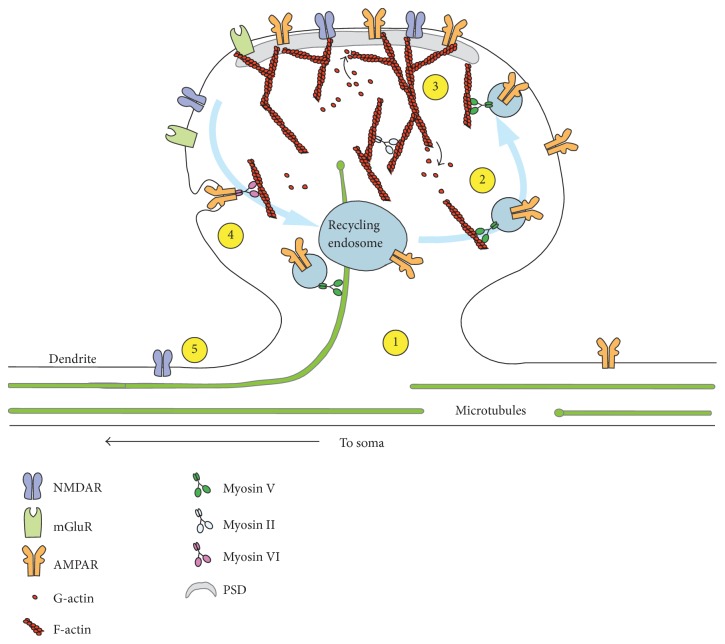
Schematic representation of the cytoskeleton-dependent trafficking of neurotransmitter receptors. Depicted are the key structures of the synapse and associated cytoskeletal molecules. Numbers indicate the following steps: (1) Myosin V traffics vesicles with receptors from the soma to the distal dendritic sites via microtubules (MT) [116]. MT plus ends are indicated by green circles. (2) Once within spines, myosin V transports receptors to plasma membrane via actin filaments [117]. (3) Anchoring of receptors in the PSD relies on myosin II contractile force on actin cytoskeleton in combination with constant actin treadmilling/turnover [48]. Lateral diffusion of receptors to and from the PSD to presynaptic regions can occur. (4) Receptor internalization involves myosin VI activity. Myosin VI transports internalized receptors to endosomal organelles, facilitating recycling of receptors back to the membrane or to degradation pathways [95]. (5) Receptors can also travel between the PSD and peripheral sites [118, 119].

Synaptic function directly correlates with the composition of neurotransmitter receptor integration at the postsynaptic membrane. The cellular and molecular mechanisms of ionotropic receptor trafficking have been extensively reviewed [113–115]; here we will focus on regulatory mechanisms by which the cytoskeleton controls the neurotransmitter receptor expression profile in excitatory synapses. See Figure 1 for a schematic summarising the mechanisms involved in glutamate receptor trafficking.

### 5.1. AMPA Receptor Trafficking

AMPARs are the primary ionotropic glutamate receptors responsible for fast excitatory synaptic transmission. At the postsynaptic membrane, regulation of AMPAR levels determines the strength of synaptic transmission. During LTP induction, AMPARs are inserted into the PSD and are removed during LTD. AMPARs comprise a combination of four subunits GluA1–4 (GluR1–4) [120] forming heterotetrameric channels [121]. However, the majority of synaptic AMPARs have GluA1/2 subunit composition [122].

AMPAR trafficking to and from the plasma membrane depends on actin dynamics. Prolonged treatment (24 h) of cultured primary hippocampal neurons with latrunculin A, an inhibitor of F-actin nucleation, attenuates the expression of GluA1 AMPAR subunit in dendritic spines [123]. Hippocampal neurons, treated with latrunculin A prior to tetraethylammonium (TEA) LTP induction, prevented insertion of GluR1 during TEA LTP. Furthermore, treatment with 1 *μ*M jasplakinolide prior to TEA also inhibited the insertion of GluR1 in response to TEA LTP induction [124]. Furthermore, treatment with jasplakinolide, an F-actin stabilizer, prevented AMPAR endocytosis during intense NMDAR activation [124]. These results suggest that expression of AMPARs at the postsynaptic membrane requires both polymerization and disassembly of the actin cytoskeleton, and F-actin stabilization is required to anchor AMPARs at the plasma membrane. Overall these studies show that AMPAR trafficking and anchoring require actin cytoskeletal dynamics and remodelling.

AMPAR trafficking processes are facilitated by actin cytoskeleton dynamics. GTPases such as Rho Ras and Rac activate downstream effectors that in turn stimulate or inhibit the activity of actin-associated proteins thereby regulating actin cytoskeletal dynamics [125]. Based on this, it is not surprising that actin-associated proteins and their upstream activators are able to affect trafficking of glutamate receptor subunits.

Vasodilator-Stimulated Phosphoprotein (VASP) regulates and modulates synaptic strength through actin polymerization. VASP has been shown to bind to both actin and profilin, promoting F-actin polymerization and preventing barbed end capping [126–128]. VASP has also been found to be essential for synapse maintenance. Overexpression of VASP increased dendritic spine volume, F-actin content, and the expression GluR1 [129]. Conversely, knockdown of VASP reduced the density of dendritic spines and synapses and GluR1 subunits within the spine [129].

Arp2/3 gates the trafficking of endosomal vesicles and internalization of AMPARs. Arp2/3 is inhibited by protein interacting with C kinase 1 (PICK1) which is in turn inhibited by the activity of GTPase ADP-ribosylation factor 1 (Arf1). Inhibition of Arp2/3 by PICK1 overexpression facilitates endocytosis of AMPAR subunits during LTD [130]. Similarly, blocking Arf1 results in AMPAR endocytosis [131].

Further studies implied a role of PICK1 in LTD. Knockdown of PICK1 prevented NMDAR LTD induced removal of GluA1 subunits from the plasma membrane. More specifically, a time course analysis of AMPAR endocytosis in response to NMDAR-mediated LTD showed that PICK1 knockdown did not prevent the initial endocytosis of AMPARs but failed to retain the receptors intracellularly. Recycling of AMPARs back to the plasma membrane during knockdown of PICK1 inhibited NMDAR-induced LTD [132].

Myosin motor proteins are critical for AMPAR trafficking. Myosin motors Va and Vb are considered efficient organelle transporters, their long lever arms allowing them to travel along the top of actin filaments in a step-like fashion rather than spiralling around the filament [133]. Interference of myosin Va and Vb function has been shown to impact neuronal cell shape and function, including changes in the composition of the PSD and modulation of LTD and LTP induction [133]. Myosin Va binds directly with the C-terminal of GluA1 subunits [117] and is required for transportation of AMPARs from the dendritic shaft into spines. Furthermore, myosin Va is implicated in the induction of LTP. Imaging of GFP-GluR1 showed that depletion of myosin Va resulted in reduced expression of GluR1 to synapses in response to CAMKII mimicked LTP. Electrophysiological experiments using siRNA knockdown of myosin Va abolished LTP induction as indicated by AMPAR mediated responses [117]. In a separate study by Wagner and colleagues [134], myosin Va was found to be critically involved in the trafficking of smooth endoplasmic reticulum (ER) into the spines of Purkinje neurons. Attenuation of myosin Va motility along actin filaments inhibited the insertion of smooth ER tubules into the spines of Purkinje neurons [134]. Reduction of ER tubule insertion minimised transient Ca^2+^-release upon mGluR activation, a process required for LTD within the cerebellum [134]. No changes were observed in fast AMPAR mediated Ca^2+^-transients. These studies suggest that myosin Va is involved in various functional aspects of synaptic plasticity, including the trafficking of smooth ER into spines and the exocytosis of AMPARs.

Myosin Vb localizes to different regions of neurons in an age dependent manner. At 7–14 days* in vitro* (DIV) this particular motor protein was detected in the soma and dendrites while being absent at synapses [116]. At more mature ages, 21–28 DIV, myosin Vb was predominantly detected in the soma but was also observed in dendritic spines that were positive for synaptic markers synaptophysin and PSD95 [116]. Lisé and colleagues suggest that these results imply that myosin Vb is responsible for the initial transportation of cargo from the soma to distant dendritic sites early on in neuronal development and then remains locally at synaptic regions where it is involved in delivery and recycling of cargo to the synapse once the neuron has matured. A more recent study by Wang and colleagues [96] also detected myosin Vb enrichment in dendritic spines using immunocytochemical techniques. In addition to this, Wang et al. also determined that myosin Vb is involved in trafficking of recycling endosomes into spines. Recycling endosomes are a source of AMPARs and are responsible for directing these receptors back to the plasma membrane during LTP induction [135, 136].

The role of myosin Vb in the transportation of glutamate receptor subunits within the postsynaptic compartment was determined using transfection of functionally deficient or dominant negative versions of myosin Vb and the small GTPase Rab11 [116]. Expression of myosin Vb C-terminal tail constructs, fused to GFP, resulted in a reduction of GluR1 subunit clustering at sites, positive for synaptophysin. This suggests that full length myosin Vb is required for delivery of GluR1 subunits to the synapse [116]. Furthermore, Lisé and colleagues found that expression of this construct did not alter the localization of GluR2/3 subunits, implying that myosin Vb may specifically regulate pools of GluR1 homomeric AMPARs. Myosin Vb is thought to associate with GluR1 through RabII coupling [116]. RabII is a recycling endosome protein that binds to myosin Vb via C-terminal amino acids 1797–1846 [137]. Neurons transfected with myosin Vb mutants that lack the C-terminal domain required for RabII binding had reduced GluR1 clustering and surface expression, suggesting that myosin Vb trafficking of GluR1 is mediated by RabII binding [116]. Furthermore, binding of RabII requires a conformational change in the myosin Vb protein that occurs in response to Ca^2+^ [96].

Unlike myosin V, myosin VI has been reported to travel along F-actin towards the pointed/minus end of the filament [138, 139]. From coimmunoprecipitate assays myosin VI has been shown to associate with GluR1 and GluR2 subunits [95]. Furthermore, myosin VI has been shown to form a complex with GluR1 and the scaffolding protein SAP97, suggesting a functional link between the actin cytoskeleton AMPAR subunits and the postsynaptic scaffold [140]. Hippocampal neurons deficient in myosin VI had greatly reduced levels of internalized AMPARs after AMPA stimulation compared to controls suggesting that myosin VI is important for endocytosis of AMPARs. In addition to this Osterweil and colleagues [95] confirmed that myosin VI trafficking of AMPARs occurs via clathrin-mediated endocytosis.

*Cofilin in AMPAR Trafficking.* Using live imaging recordings, Gu et al. [141] found that inhibition of LIMK1 resulted in enhanced trafficking of GluR1 and inhibition of SSH resulted in diminished GluR1 trafficking to the spine surface during TEA-induced LTP. Due to the relationship between LIMK1 and SSH and cofilin, these results suggest that cofilin activation is required for expression of AMPARs during LTP. As there were no significant changes in spine head to neck ratios before and after 6 h treatment with LIMK1 and SSH inhibiting peptides, Gu and colleagues concluded that cofilin mediates AMPAR trafficking independent of the actions of cofilin on cytoskeletal spine structure. Consistent with this finding, Yuen and colleagues [142] also found a reduction in AMPAR excitatory postsynaptic current (EPSC) amplitude and frequency in response to siRNA knockdown of SSH.

In an experiment investigating the role of cofilin in an aversive conditioning paradigm, it was observed that cofilin was temporally enhanced in the infralimbic cortex of rats after extinction of an aversive memory [143]. Furthermore, elevation of cofilin levels resulted in an increase in surface expression of GluA1 and GluA2 subunits and facilitated extinction learning. Conversely, inhibition of cofilin during extinction training prevented the insertion of these subunits at the plasma membrane and impaired extinction learning [143].

These studies imply that the F-actin severing properties of cofilin are required for AMPAR recruitment at the plasma membrane during LTP. This finding would fit well with the suggested cofilin mediated cytoskeletal reorganization during LTP posed by Chen and colleagues [58] whereby cofilin severing of F-actin allows for enlargement of dendritic spine volume during LTP. As AMPAR expression at the postsynaptic membrane and spine volume are tightly correlated it is plausible that cofilin activity mediates these processes. 

*Drebrin in AMPAR Trafficking.* Electrophysiological recordings demonstrated that expression of DA-GFP in neurons enhances excitatory transmission compared to GFP controls. Inhibition of drebrin A by DA antisense oligonucleotides further supported a role of DA in synaptic transmission as inhibition of DA resulted in a decrease in excitatory transmission, indicated by decreases in miniature EPSC (mEPSC) amplitude and frequency, compared to controls [71]. Drebrin knockdown was found to impair AMPAR mediated mEPSC amplitude and frequency in hippocampal neurons. In addition, mEPSC amplitude and frequency in response to glutamate-induced LTP were reduced during drebrin knockdown. These results suggest that drebrin is involved in AMPAR trafficking and insertion at the plasma membrane [144]. 

*4.1N in AMPAR Trafficking.* Protein 4.1N is a homolog of 4.1R, a protein found to be an integral component of the cytoskeleton in erythrocytes. 4.1N has been found to localize in various regions of the brain including the CA1–CA3 areas of the hippocampus [145]. Furthermore, immunocytochemical analyses showed colocalization of 4.1N with PSD95, suggesting that 4.1N localizes to sites of synaptic connection [145]. Like its erythrocyte homolog, 4.1N is thought to associate with the actin cytoskeleton [145].

Using coimmunoprecipitation and deletion of various amino acids in GluR1 proteins, 4.1N was found to associate with GluR1 at the membrane proximal region of the C-terminal domain at amino acids 812–823 [146]. Truncations of GluR1 at this membrane proximal region resulted in GluR1 becoming incapable of associating with 4.1N and having decreased expression at the plasma membrane [146] suggesting that 4.1N activity is required for expression of GluR1 subunits in the plasma membrane.

### 5.2. NMDA Receptor Trafficking

N-Methyl-D-aspartate receptors (NMDARs) are glutamate receptors, present in smaller numbers than AMPARs, which serve to modulate excitatory transmission by affecting AMPAR expression [113]. Activation of AMPARs leads to removal of the Mg^2+^ block of NMDARs, allowing for calcium influx. This calcium influx affects a variety of pathways which regulate the expression of AMPARs.

NMDARs have a heterotetrameric structure, usually consisting of two GluN1 and two GluN2 subunits. The subunits are produced and assembled in the endoplasmic reticulum in the cell body and then moved to the spine by various kinesins, moving along microtubules [113]. While transport to the spine involves only microtubules and not actin, short distance transport in the spine head to the PSD seems to be dependent on actin and myosin motor proteins [147]. However, the exact proteins and mechanisms involved are still unclear. Myosin IIb has been shown to be involved in insertion of NMDARs at the membrane, but it is not believed that it is responsible for transporting it to the membrane [148]. At the surface of the synapse, NMDARs are associated with scaffolding proteins of the PSD [149].

Endocytosis of NMDA receptors can be triggered by low-frequency stimulation [150]. Receptor clusters can move laterally in and out of the synaptic site. When NMDARs were irreversibly blocked with a drug, recovery of NMDAR-mediated current was observed, suggesting receptors migrated to the synaptic site from the periphery [118]. Actin dynamics have also been shown to affect NMDAR placement at the synapse [123].

There is evidence that actin dynamics have a regulatory role in NMDAR function. Alpha-actinin-2, an actin binding protein present in the PSD, competes with calmodulin for binding to NMDARs [151]. Alpha-actinin is a protein that binds to both NMDARs [152] and components of the PSD [153] and has a role in spine morphology [154].

Severing of actin filaments or preventing polymerization of actin has been found to induce a rundown of NMDAR current. Inactivation of RhoA which promotes actin polymerization increases rundown of NMDAR current [155]. Similarly, administering cytochalasin, a drug blocking actin polymerization, also induces rundown [156]. Conversely, knockout of gelsolin enhances NDMAR current [157]. Modification of actin can also affect placement of receptors in the postsynapse. Latrunculin A, an inhibitor of actin polymerization, modifies localization of NMDA receptors [123].

Another way actin may be involved in NMDAR trafficking is through its function in microtubule extension. Drebrin, an actin stabilizing protein, interacts with both actin microfilaments and microtubules and promotes entry of microtubules into dendritic spines [158]. NMDARs also have a part in mediating spine morphology. Deletion of the NR1 subunit has been found to reduce spine density and increase head size [159]. The actin severing protein cofilin is required in spines for NMDAR-induced remodelling [160].

### 5.3. mGluR Receptor Trafficking

As well as the ionotropic glutamate receptors, there are also metabotropic receptors (mGluRs) present at the postsynaptic membrane. Unlike NMDARs and AMPARs, metabotropic receptors are G-protein coupled receptors. They produce their effects through signalling pathways involving inositol phosphate (IP), diacylglycerol (DAG), activation of protein kinase C (PKC), and release of intracellular Ca^2+^ stores. There are three families of mGluRs: Groups I, II, and III. In neuronal tissue, Groups II and III are located on the presynaptic membrane, while Group I is located on the postsynaptic membrane. Group I includes mGluR1 and mGluR5. They are comprised of GluA1 and GluA5 receptor subunits. Little is known about synthesis and trafficking of mGluRs to the spine, but there is evidence that, like NMDARs, they are associated with the PSD [161].

mGluRs can move laterally on the postsynaptic membrane. It has been found that transport of mGluR5 on the membrane surface involves being bound to microtubules, and the movement of these microtubules was dependent on actin flow. Preventing actin polymerization through application of cytochalasin D disrupted the movement of mGluR5 on the membrane [119]. In cultured hippocampal neurons, mGluRs have been found to be located at perisynaptic regions of excitatory synapses [162]. mGluR function can be modified by signalling molecules. One example is Rab8, a small GTPase involved in vesicular trafficking. It binds to the C-terminal tail of mGluRs and inhibits the production of IP and the release of intracellular Ca^2+^ [163] Rab8 expression resulted in inhibition of mGluR1 endocytosis, maintaining cell surface expression of the receptors [163]. mGluRs are responsible for activity-dependent synaptic plasticity through their role in regulating trafficking of other glutamate receptors. It has been found that blocking both mGluR1 and mGluR5 prevented induction of LTD, suggesting that mGluRs have a role in mediating AMPAR endocytosis [164].

## 6. Disruption of the Actin Cytoskeleton and Neurotransmitter Receptor Trafficking in Disease

While current research on pathological mechanisms of AD encompasses the study of a diverse range of potential mechanisms, a central theme underlying AD pathology is the loss of synaptic connectivity. Neural connections within the brain underlie the most basic and fundamental requirements for successfully interacting with the world around us. The loss of these neural circuits can catastrophically impair one's ability to function independently, as observed in AD. Although various gene mutations have been implicated in familial forms of AD [165] the causes behind the onset of AD pathology are as yet unknown.

A considerable effort in Alzheimer's disease research has been to identify the brain regions most vulnerable to degeneration. Over the last two decades the literature has reported significant hippocampal deterioration in early AD pathology [1, 166–169]. Hippocampal volume is often used as a diagnostic tool for AD as the level of deterioration positively correlates with the severity of AD symptoms [170, 171]. Other features of the medial temporal lobe, wherein lies the hippocampus, such as cortical thickness, have also been described as reliable indicators of AD pathology [172–174]. These hippocampal measures have also been successful as an indicator of AD vulnerability in presymptomatic patients [175, 176].

The molecular mechanisms of AD pathogenesis are still not well understood. The two major pathological hallmarks of AD are the extracellular accumulation of proteolytic derivations of amyloid precursor protein (APP) called amyloid-*β* (A*β*) peptides and intracellular aggregation of tau protein fibrils. Accumulation of these abnormal proteins is thought to be responsible for the breakdown of synapses, decreases in spine density, and impairment of synaptic plasticity [177–179]. Although studies have highlighted interplay between these two pathological markers [180–182], evidence suggests that the accumulation of A*β* oligomers accelerates tau aggregation and synaptic loss [183, 184]. The biosynthesis of A*β* arises as a residual product from *α*-secretase failing to cleave APP [185]. In the absence of *α*-secretase activity, *β*- and *γ*-secretases cleave APP, generating A*β* peptides. Large oligomeric peptides have been shown to be neurotoxic in comparison to small A*β* oligomers and soluble monomers, which have been implicated in neuroprotective processes [186, 187].

### 6.1. Pathological Role of Soluble and Aggregated Forms of Amyloid *β* Peptide

Amyloid fibrils are aggregations of long, insoluble fibres of A*β* peptide. Protofibrils (shorter, soluble aggregations that are precursors to fibrils) and fibrils have been observed to have an overexcitatory effect on neurons and interactions with NMDA receptors [188]. Amyloid plaques, large extracellular deposits of A*β* fibrils, are the most obvious form of pathophysiology, associated with Alzheimer's disease. Behavioural deficits of dementia have previously been found to be correlated with the size of the cortical area affected by plaques [189]. In the study by Cummings and Cotman, deposition of A*β* was found to strongly correlate with scores on the Mini-Mental State Exam (MMSE), the Blessed Information Memory Concentration (IMC) test, and Clinical Dementia Rating (CDR) with higher deposition resulting in poorer scores. In brain slice cultures, plaque-covered areas contained only few dendritic spines and spine volume was reduced in the areas around the plaque [190]. However, synaptic deficits can occur in the absence of plaques [191] and the extent of plaque formation does not always correlate with the degree of neurodegeneration or clinical status of AD [192, 193]. More recent studies are still debating as to whether or not plaque formation is responsible for behavioural deficits in AD. In some of these studies, behavioural deficits could be rescued in response to a reduction in amyloid plaques [194, 195]. However, these studies also observed reductions in soluble A*β*, which is believed to be highly toxic when in oligomeric form [187]. Cramer and colleagues [196] used a mouse model of AD to show that increasing levels of apolipoprotein E (apoE) can lead to a reduction of soluble and insoluble A*β*. ApoE is involved in the proteolytic degradation of soluble forms of A*β*. Acute increases in apoE resulted in significant reductions in both soluble A*β* and plaque quantity. AD-associated learning deficits were also reduced in both the Morris water maze and contextual fear conditioning paradigms upon treatment with apoE [196]. However, these behavioural measurements were found to only correlate with reduced levels of soluble A*β* and not with plaque removal. Plaque formation can be present without cognitive decline [197]. This shifted the focus to an increased interest in understanding the pathological role of soluble forms of A*β*. Soluble A*β* forms include monomers, dimers, and larger oligomers of A*β* protein. Soluble A*β* is localized to the postsynaptic compartment in both animal models of Alzheimer's disease [198] and human patients [199]. A*β* oligomers cause alterations to pre- and postsynaptic morphology, including spine shrinkage and collapse [200]. LTP has been found to be inhibited in brain slices after application of oligomers sourced from cell cultures [201], dimers extracted from human AD brains [178], and synthetic A*β* oligomers [202]. A*β* can also facilitate LTD. Application of oligomers from different sources allowed LTD to be induced in conditions that are normally insufficient to do so [203].

### 6.2. The Effects of Amyloid *β* on the Regulation of the Actin Cytoskeleton

**Figure 2:**
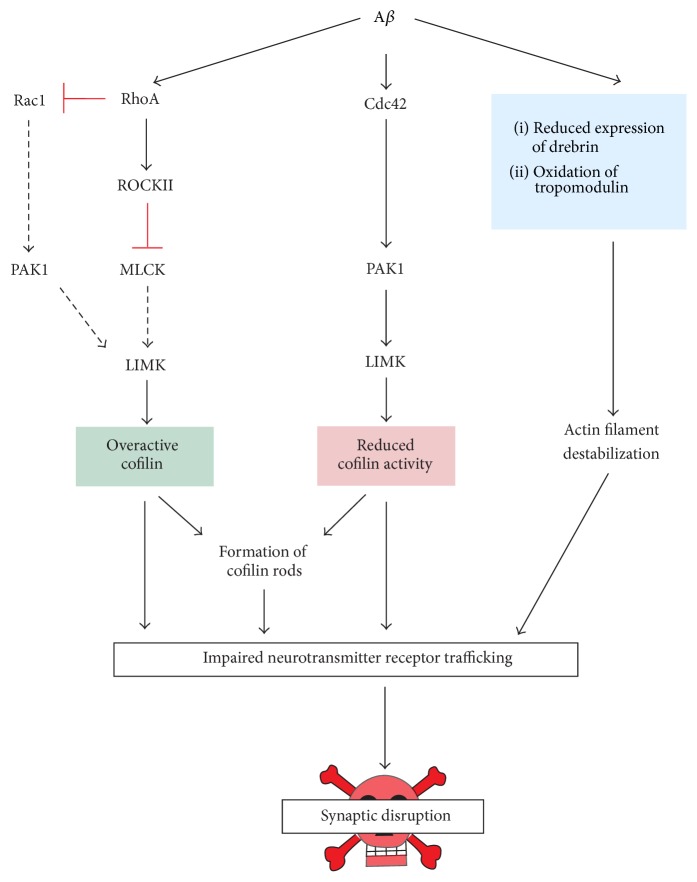
Amyloid-*β* disrupts the actin cytoskeleton and receptor trafficking through multiple pathways. There are many conflicting pathways through which A*β* is proposed to alter the actin cytoskeleton. These may involve both up- and downregulation of cofilin activity. Activation of RhoA by A*β* [204] antagonistically inhibits Rac1 [206], both leading to increased cofilin activity. In contrast, A*β* can cause decrease in cofilin activity via activation of Cdc42 [211]. Both active and inactive cofilin are thought to be required for the formation of cofilin rods, which lead to impairment of intracellular transport [215]. Alternatively or in addition to this, altered expression and/or processing of actin filament stabilizing proteins [217, 218] may impact the trafficking of neurotransmitter receptors.

There are many conflicting pathways in which A*β* is proposed to alter actin cytoskeletal dynamics. The predominant theories involve modulation of cofilin activity. Conflicting evidence is found throughout the literature that suggests that cofilin is either activated or inhibited in response to A*β* toxicity (see Figure 2). Petratos and colleagues [204] reported an increase in active RhoA in SH-SY5Y cells treated with A*β*. RhoA activates Rho kinase II (ROCKII), which leads to the deactivation of myosin light chain kinase, dephosphorylating and thereby inhibiting the actions of LIMK [205]. Another pathway in which A*β* is proposed to increase cofilin activation is through inhibition of Rac1. RhoA activation requires deactivation of Rac1 [206]. Therefore it is suggested that A*β* induced increases of RhoA antagonistically decrease levels of Rac1. Rac1 inhibition reduces PAK1 signalling, which reduces the phosphorylation and activation of LIMK [207, 208]. This pathway is supported by findings of decreased PAK1 in the brains of AD patients [209]. Inactivation of LIMK in both pathways would result in reduced phosphorylation and as such the activation of cofilin [56]. Increased activation of cofilin may then disrupt receptor trafficking through disassembly of the actin cytoskeleton [208] and/or formation of cofilin rods [210]. Conversely, various studies suggest that A*β* ultimately inhibits cofilin activation through alternate signalling pathways. Mendoza-Naranjo and colleagues [211] found an increase in levels of GTPase Cdc42 in hippocampal neurons treated with fibrillar A*β*. Cdc42-PAK1-LIMK signalling cascades result in decreased cofilin activation [56, 212] which would have implications for actin cytoskeleton dynamics.

The formation of cofilin rods, abnormal aggregates of bound actin and cofilin, has been shown to disrupt vesicle transport and cause accumulation of A*β* and APP [210, 213, 214]. Blocking intracellular trafficking by cofilin aggregation induces synaptic loss in hippocampal neurons [215]. Hippocampal neurons transfected with wild-type GFP-cofilin resulted in the formation of cofilin-actin rods. Transfections using cofilin mutants, phosphomimetic GFP-cofilin and constitutively active GFP-cofilin, resulted in no or reduced cofilin rod formation, respectively. This suggests that both active and inactive cofilin are required for the formation of cofilin rods, a requirement potentially fulfilled by the contradicting pathways mentioned above. As these mutations involved the phosphorylation Ser3 site on cofilin it is believed that the formation of cofilin rods is critically dependent on this site [215]. Immunostaining detected rod localization in distal dendrites and occasionally in axons. An additional observation was made in that decreased MAP2 fluorescence was apparent in regions containing rod formation compared to neighbouring regions absent of rods. The authors suggest that this could imply impairment of microtubule integrity [215]. Formation of cofilin rods from endogenous levels of cofilin was observed in response to glutamate treatment or neurotoxic ATP depletion [215].

Immunostaining of RFP-cofilin and GFP-Rab5, a small GTPase that localizes to early endosomes [216], showed that, in areas where cofilin rods appeared, early endosomes positive for Rab5 were either largely absent or positioned at the distal or proximal ends of rods suggesting that they were immobilised at these regions. This finding was similar for imaging of GFP-mitochondria, where localization was found to be either largely absent in regions containing rods or trapped between rods. These results imply that rod formation disrupts intracellular organelle distribution [215]. This was further confirmed using live imaging techniques. Prior to rod formation, mitochondria appeared to be able to freely move within the cell; however, after rod formation mitochondria trajectory was significantly restricted and slower than that before rod formation [215].

Rod formation was also found to affect synaptic transmission and induce synaptic loss. Markers of pre- and postsynaptic regions were significantly reduced in areas containing rods. In addition to this, dendritic spine density was also decreased in rod rich areas. The cofilin rod reduction in spine density was in part supported by electrophysiological recordings of hippocampal neurons expressing varying quantities of cofilin rods. A significant reduction in mEPSC frequency but not amplitude was observed in neurons with exceptionally high levels of rods [215]. Neurons with mild levels of rods had mEPSC frequencies and amplitudes that were comparable to controls. These results suggest that aggregation of cofilin rod formation induces synaptic loss, which eventually leads to the loss of neuronal function [215].

Decreased levels of gelsolin were found in AD patients' plasma. The level of decrease was correlated to progression of disease, as measured with a Mini-Mental Status Examination [219]. Gelsolin has also been found to be involved with removal of A*β*. Gelsolin forms a complex with A*β*, making it less neurotoxic [220].

### 6.3. The Effects of Amyloid *β* on the Trafficking of Neurotransmitter Receptors

It is plausible then that neurotoxic stimulation via soluble A*β* activity drives the formation of cofilin rod formation in AD pathology and through this pathway disrupts synaptic properties such as AMPAR trafficking via early endosomes. AMPARs can be internalized to early endosomal organelles where they are then transferred to recycling endosomes for reinsertion at the plasma membrane, or to late endosomes for degradation depending on the type of synaptic stimulation [221]. AMPAR stimulation induces endocytosis of AMPARs to early endosomes followed by transferral to late endosomal organelles and subsequent degradation [135, 221]. NMDAR excitation on the other hand results in AMPAR internalization to early endosomes and then to recycling endosomes ultimately leading to the reinsertion at the plasma membrane [135, 221]. As A*β* is thought to weakly activate NMDARs [222], it is postulated that AMPARs are endocytosed to early endosomes but are possibly unable to be transferred to recycling endosomes due to cofilin-actin rod obstructions. This would result in weakening and eventual loss of synapses as is observed in both neurons with extensive cofilin-actin rod formation and also neurons afflicted by A*β*. Recent research confirms the importance of recycling endosome location on synaptic potential. Positioning of endosomes has been found to be important for AMPAR trafficking and synapse architecture, with the removal of recycling endosomes from the spine resulting in decreased surface AMPAR levels [223].

A potential way by which synapses are destabilized is the loss or functional disruption of actin stabilizing proteins at the synapses. Drebrin has been found to be reduced in the brains of AD and Down syndrome patients [217]. Drebrin reduction is also associated with impaired synaptic plasticity [224] and altered movement of NMDAR clusters to synapses [225]. In brains from AD cases, Tpms were found in neurofibrillary tangles (NFT), intracellular protein aggregates of abnormally phosphorylated tau protein [226, 227]. However, studies are limited, because antibodies used were not specific for Tm isoforms: identities of specific isoforms in NFTs are unknown. Interestingly, Tpm3.1 is a major target of oxidative damage in AD, suggesting that disruption of Tpm3.1 may contribute to pathological changes in the disease [218]. Pathways by which A*β* affects the nucleation of actin filaments may be closely connected to those disrupting microtubule dynamics. Knockdown of the formin mDia1 in NIH3T3 cells reduces A*β* induced pathological stabilization of MTs [228].

## 7. Conclusions

In this paper we discussed the current understanding of the role that the actin cytoskeleton plays in the regulation of the postsynaptic compartment, how it drives structural changes, how it supports neurotransmitter receptor trafficking and synaptic function, and how these processes are disrupted in neurodegenerative diseases. Currently, there are no efficient treatments for stopping or even reversing the pathological mechanisms in neurodegenerative diseases such as AD. A more detailed understanding of the regulatory mechanisms of the postsynaptic cytoskeleton may allow us to develop new strategies for protecting synaptic connections and to increase their resistance to pathological effects in the disease. In particular, it remains to be fully understood how the trafficking of glutamate receptors is disrupted by the presence of A*β*. As many actin-associated proteins exist in the cell in antagonistic relationships with other actin-associated proteins, it would be interesting to know the extent to which alteration of one regulatory protein affects others. Furthermore, studies involving* in vivo* techniques would provide a more accurate picture of how actin cytoskeleton dynamics influence the trafficking of AMPARs and ultimately synaptic plasticity. In addition to these studies, new advances in superresolution imaging could be implemented to examine not only the mobility of AMPARs in response to alterations in various actin-associated proteins but also changes in the distribution and localization of these proteins. Eventually, this leads us to the question of whether we can develop strategies that target specifically the synaptic actin cytoskeleton* in vivo*. Most actin cytoskeleton targeting drugs are rather unspecific for the actin filament populations that are manipulated. More recent approaches have shown that specific subpopulations of actin filaments can now be directly manipulated [229]. To exploit the use of these drugs for the potential therapeutic use in treating neurological disease, a detailed understanding of how different actin filament populations at synapses are formed, maintained, and turned over will be essential considerations for future studies.
